# Recurrent Syncope following Substance Abuse; a Case Report

**Published:** 2017-01-14

**Authors:** Forod Salehi, Mohammad Mehdi Hassanzadeh Taheri, HamidReza Riasi, Omid Mehrpour

**Affiliations:** 1 Department of Pediatric, Birjand University of Medical Sciences, Birjand, Iran.; 2 Department of Anatomical Sciences, Faculty of Medicine, Birjand University of Medical Sciences, Birjand, Iran.; 3 Neurology Department, Birjand University of Medical sciences, Birjand, Iran.; 4 Atherosclerosis and Coronary Artery Research Center, Birjand University of Medical Sciences, Birjand, Iran.; 5 Medical Toxicology and Drug Abuse Research Center, Birjand University of Medical Sciences, Birjand, Iran.

**Keywords:** Substance-related disorders, syncope, amphetamine, N-Methyl-3, 4-methylenedioxyamphetamine, case report

## Abstract

Drug abuse is considered as the most common poisoning in the world. Stimulants agent especially amphetamines and methamphetamines are among important abused substances. Different types of neurologic, psychiatric, respiratory, gastrointestinal, and cardiogenic complications have been reported to be related to methamphetamine consumption. Some of these substances could cause dysrhythmias which is the most prevalent etiology of cardiogenic syncope. Ecstasy, as one of the most commonly abused drugs, is known as a cause of cardiac dysrhythmias. Here we report a young boy who was admitted into the emergency department following three syncope attacks. All cardiac and neurologic assessments were normal; and finally ecstasy abuse was detected as the main etiology of syncopes.

## Introduction

Drug abuse is considered as the most common poisoning in the world and about 2 to 5 million of such poisoning occur annually in the united states (1). Stimulants agent especially amphetamines and methamphetamines are among important abused substances (2). Different types of neurologic, psychiatric, respiratory, gastrointestinal, and cardiogenic complications have been reported to be related to methamphetamine consumption (-). Some of these substances could cause dysrhythmias which is the most prevalent etiology of cardiogenic syncope (8). To emphasize the importance of this topic, here we report a case of recurrent syncope following amphetamine abuse.

## Case presentation:

**Figure 1:**
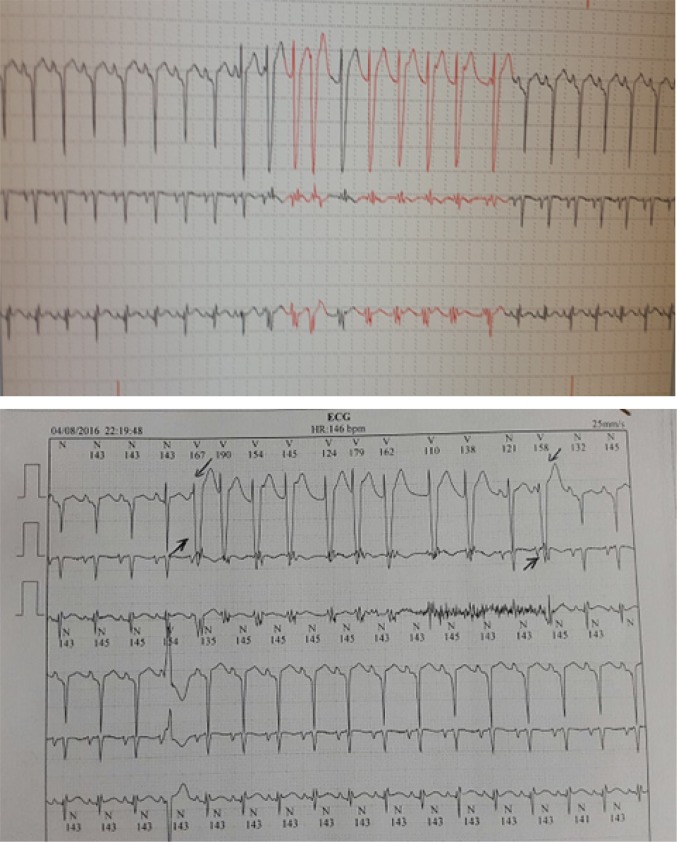
24 hours cardiac holter monitoring. a uniform, sustained ventricular tachycardia.

An 18-year-old boy was admitted to the emergency department of Markaze–Tebi–Koodakan Hospital, Tehran, Iran with chief complaint of sudden weakness, transient loss of consciousness and falling down at home. A meticulous history of the patient revealed 2 similar attacks in last 3 months. Further evaluations in previous attacks including brain imaging, cardiac stress test were all negative and genetic testing revealed no evidence of channelopathies. There was no history of head trauma, cardiac disease or regular medication use. The patient denied any substance abuse and family history revealed no sudden cardiac death. The patient's vital signs on admission were stable, neurological and cardiac examinations were normal. His Glasgow coma score (GCS) was 15/15, pupils responded normally to light, deep tendon reflex (DTR) and cranial nerves examinations did not revealed any abnormality. Electrolytes, blood sugar level, and thyroid function tests were performed and all were reported in normal range. The patient's electrocardiogram (ECG) on arrival was normal. Echocardiographic evaluation showed no structural heart disease. Brain magnetic resonance imaging (MRI) and electroencephalography (EEG) were normal and the tilt test result was negative. Twenty-four hour cardiac holter monitoring was performed in which a uniform, sustained ventricular tachycardia (VT) was revealed (Figure 1). Psychiatric consultation was carried out due to poor family support and chaotic family interactions. Psychiatric consultation revealed that the patient had two previous suicide attempts and history of ecstasy abuse. Based on history, he had used ecstasy a night before, thereby resulting in syncope attacks. An evaluation of serum toxins level revealed a methamphetamine serum level of 12 mg/dl. The patient was discharged with a diagnosis of syncope caused by VT due to methamphetamine abuse. Psychiatric follow-up was advised for 6 months. During this period, ecstasy usage was discontinued and he did not experience any episode of syncope attacks.

## Discussion

Although, intoxication and their complications are usually associated with overdose consumption of abuse drugs, but the use of actual dose of amphetamine may lead to cardiovascular events (9). Palpitation, premature ventricular and supraventricular contraction, accelerated atrioventricular conduction, atrioventricular block, bundle branch block, supraventricular tachycardia, ventricular tachycardia and fibrillation are among the most prevalent cardiogenic complications (10). In a study by Fabrizio et al. it was shown that methylenedioymethamphetamine (MDMA) induces arrhythmia through the release of serotonin and catecholamine (especially noradrenaline), which are responsible for most severe accidents in the cardiovascular system (4). Elevated catecholamine level causes tachycardia and hypertension that lead to increased oxygen demand and vasospasm. Myocardial ischemia occurs in response to decreasing oxygen supply and increasing oxygen demand in the myocardium that leads to increasing the potential risk of cardiac arrhythmia (11). Zhuo et al. demonstrated the mechanism of reduction of connexin 43 and N-cadherin (myocardial gap junction proteins) in the pathophysiology of cardiovascular arrhythmia due to MDMA exposure. MDMA reduces both connex in 43 and N-cadherin. These forms of proteins are multiprotein complexes that could allow the assemblage of both gap and fascia adherens junctions. Loss or decreased gap junction-proteins may disrupt cardiac impulse propagation and result in ventricular arrhythmia (12).

After cannabis, methamphetamines and their compounds have become the most widely abused illicit drugs all over the world (8, 13). Ecstasy is an easily available drug and used mainly by young individuals in parties. This case study presents a young man with recurrent syncope further diagnosed as ecstasy abuse as the main cause. In syncope with unknown etiology, history of the patient must be suspected and accurately examined. 

## References

[1] Litovitz TL, Klein-Schwartz W, Rodgers GC, Cobaugh DJ, Youniss J, Omslaer JC (2002). 2001 Annual report of the American Association of Poison Control Centers toxic exposure surveillance system. The American journal of emergency medicine.

[2] Mehrpour O (2012). Methamphetamin abuse a new concern in Iran. DARU Journal of Pharmaceutical Sciences.

[3] Albertson TE, Derlet RW, Van Hoozen BE (1999). Methamphetamine and the expanding complications of amphetamines. Western Journal of Medicine.

[4] Schifano F, A bitter pill (2004). Overview of ecstasy (MDMA, MDA) related fatalities. Psychopharmacology.

[5] Lewis DA, Dhala A (1999). Syncope in the pediatric patient: the cardiologist's perspective. Pediatric clinics of North America.

[6] Liechti ME, Kunz I, Kupferschmidt H (2005). Acute medical problems due to Ecstasy use Case-series of emergency department visits. Swiss medical weekly.

[7] Al Shehri MA, Youssef AA (2015). Acute myocardial infarction with multiple coronary thromboses in a young addict of amphetamines and benzodiazepines. Journal of the Saudi Heart Association.

[8] Wijetunga M, Seto T, Lindsay J, Schatz I (2003). Crystal methamphetamine‐associated cardiomyopathy: tip of the iceberg?. Journal of Toxicology: Clinical Toxicology.

[9] Olfson M, Huang C, Gerhard T, Winterstein AG, Crystal S, Allison PD (2012). Stimulants and cardiovascular events in youth with attention-deficit/hyperactivity disorder. Journal of the American Academy of Child & Adolescent Psychiatry.

[10] Vearrier D, Greenberg MI, Miller SN, Okaneku JT, Haggerty DA (2012). Methamphetamine: history, pathophysiology, adverse health effects, current trends, and hazards associated with the clandestine manufacture of methamphetamine. Disease-a-Month.

[11] Karlovšek MZ, Alibegović A, Balažic J (2005). Our experiences with fatal ecstasy abuse (two case reports). Forensic science international.

[12] Zhuo L, Liu Q, Liu L, Sun T-y, Wang R-s, Qu G-q (2013). Roles of 3, 4-methylenedioxymethamphetamine (MDMA)-induced alteration of connexin43 and intracellular Ca 2+ oscillation in its cardiotoxicity. Toxicology.

[13] Won S, Hong RA, Shohet RV, Seto TB, Parikh NI (2013). Methamphetamine‐Associated Cardiomyopathy. Clinical cardiology.

